# Research on the influence of physical activity on procrastination behavior of medical postgraduates

**DOI:** 10.3389/fpsyg.2026.1786157

**Published:** 2026-04-16

**Authors:** Guoxing Zhang, Feng Tai

**Affiliations:** Liaoning Normal University School of Physical Education, Dalian City, Liaoning, China

**Keywords:** medical postgraduates, physical activity, positive psychological capital, procrastination behavior, self-control

## Abstract

**Objective:**

To explore the influence of physical activity on the procrastination behavior of medical postgraduates, and the chain mediating effect of positive psychological capital and self-control, and to verify the effectiveness of physical activity in improving procrastination behavior.

**Methods:**

Taking 673 medical postgraduates as the research object, the “Physical Activity Scale”, “Procrastination Behavior Scale”, “Positive Psychological Capital Scale”, and “Self-Control Scale” were used for psychological measurement. SPSS 27.0 software was used for correlation analysis, and PROCESS 4.2 plug-in was used to test the chain mediating effect.

**Results:**

(1) Physical activity can significantly negatively predict procrastination behavior, and the direct predictive effect of physical activity on procrastination behavior of medical postgraduates is significant. (2) Physical activity can significantly positively predict positive psychological capital and self-control; positive psychological capital significantly positively predicts self-control, and significantly negatively predicts procrastination behavior; self-control can significantly negatively predict procrastination behavior; (3) Positive psychological capital and self-control have a significant mediating effect between physical activity and procrastination behavior of medical postgraduates. Among the three mediating paths, physical activity → positive psychological capital → procrastination behavior (Ind1), physical activity → self-control → procrastination behavior (Ind2) and physical activity → positive psychological capital → self-control → procrastination behavior (Ind3) accounted for 23.23%, 14.65%, and 9.09% of the total effect, respectively.

**Conclusion:**

There is a significant negative correlation between physical activity and procrastination behavior of medical postgraduates. Physical activity can not only have a simple mediating effect on procrastination behavior through positive psychological capital and self-control, but also affect the procrastination behavior of medical postgraduates through the chain mediating effect of positive psychological capital and self-control.

## 1 Introduction

In the context of higher education in China, medical postgraduates are full-time college students at the postgraduate level, serving as the key link between undergraduate and doctoral education, and as the core force in cultivating high-level medical talents to promote the development of medical and health undertakings. Procrastination behavior has become a common psychological and behavioral issue among medical postgraduates. This study takes the Theory of Planned Behavior (TPB) as the main theoretical framework. TPB is a social psychology theory applied to predict and explain human behavioral intentions (Vengrin, 2016), this theory holds that behavioral intention is the most direct antecedent variable of actual behavior, and behavioral intention is determined by three core constructs: individual’s attitude towards the implementation of the behavior, perceived subjective norms, and perceived behavioral control. Therefore, TPB has become one of the main theoretical models to study the different variables that affect the practice of physical activity (Tirado González et al., 2012), which can help researchers to clarify the impact of physical activity on procrastination behavior. Emotional exhaustion, depersonalization, and a decrease in personal achievement are the three main dimensions of procrastination behavior. At present, procrastination behavior has generally become a psychological and behavioral problem among medical postgraduates, this is not only a personal problem of medical postgraduates, but also a chronic response to learning or work pressure, which in turn reduces the efficiency of learning or work. Procrastination behavior is an irrational behavior in which individuals voluntarily delay completing a task. However, they will foresee the adverse consequences of procrastination (Kirchner-Krath et al., 2025), which is usually manifested as deliberate avoidance of the task, active delay, and firm belief that there is still time to complete the task in the future, with an irrational tendency to delay. Previous studies have shown that many college students have complex causes and different degrees of procrastination. It involves not only internal psychological factors such as fear of failure and time management disorders, but also external environmental factors such as academic pressure and environmental temptation, which pose a threat to academic, emotional, and adaptive ability (Serrano Garcés et al., 2025), it fully shows that there is a significant negative correlation between procrastination behavior and learning. First of all, the empirical data show that 29.25% of the medical survival in serious academic procrastination behavior, and procrastination behavior and academic performance was significantly negatively correlated (Hayat et al., 2020); more surveys show that up to 96.1% of medical survival in different degrees of procrastination behavior (Wu et al., 2015). Secondly, the unique and high-intensity stress environment endured by medical graduate students is fertile ground for procrastination behavior, their academic course content is numerous (Morcos and Awan, 2022), and students generally feel “never finished” (Hill et al., 2018), studies have confirmed that this extreme academic requirements and competitive atmosphere lead to the psychological pressure of medical students (Rafique et al., 2019), the prevalence of depression and anxiety is significantly higher than that of non-medical students and the general population (Rotenstein et al., 2016; Cunha et al., 2023). Finally, the extreme difficulty of time management is the key mechanism to connect high-pressure environment and procrastination behavior, medical postgraduates often face serious time constraints due to heavy clinical and academic tasks, which is regarded as the primary obstacle to carry out research activities (Alyousefi et al., 2023; Prahladh et al., 2024), bad time management will directly lead to emotional distress and procrastination behavior, and is significantly negatively correlated with procrastination behavior. Therefore, there is a good reason to choose medical postgraduates as the research object, they not only have a high level of procrastination behavior, but also have overloaded academic requirements, special emotional challenges and severe time management dilemmas, which aggravate the psychological motivation of procrastination behavior.

When discussing the relationship between physical activity and procrastination behavior, existing studies have shown that regular physical activity can directly and indirectly reduce procrastination behavior, and its mechanism of action is often achieved by improving key psychological resources such as individual self-control, self-efficacy and self-esteem (Li et al., 2022), these psychological resources do not exist in isolation, and they together constitute a more macro and integrated positive psychological construct-positive psychological capital. Physical activity is a kind of positive lifestyle, which has been paid more and more attention to for the improvement of psychological and behavioral problems. Empirical research has preliminarily revealed that there is a significant relationship between physical activity and procrastination behavior (Xielin et al., 2025), and there is a direct negative correlation. For example, the procrastination level of students participating in group sports such as football is generally significantly lower than that of non-athletes (Ahmad et al., 2024). At the same time, it can indirectly reduce procrastination behavior by creating a positive physical activity atmosphere (Dong et al., 2025). Long-term, systematic, and regular physical activities can effectively improve the tendency of students’ procrastination behavior, significantly enhance the individual’s awareness of time monitoring, so that they can plan tasks more reasonably and improve execution efficiency, thus reducing procrastination behavior. In addition, physical activity can also intervene in mental state (Gan et al., 2025) and promote the use of positive emotion regulation strategies, reduce the negative emotions caused by task aversion, and then improve the willingness to perform tasks (Herzog-Krzywoszanska et al., 2024). However, the path of physical activity to reduce procrastination behavior through what psychological mechanism needs to be further analyzed.

Positive psychological capital covers four dimensions: self-efficacy, hope, resilience, and optimism. It has been proven to be an effective protective factor for procrastination and can negatively predict the level of procrastination (Yin et al., 2023). Positive psychological capital is an individual’s internal positive psychological resource, which is the individual’s ability to find a variety of ways to achieve success (Su and Hahn, 2023). It may directly negatively predict procrastination behavior, or it may reduce procrastination behavior by enhancing the individual’s ability to self-control. In addition, self-control, as an individual’s ability to overcome impulses and regulate behavior to meet long-term goals, is widely considered to be the most direct psychological proximate cause of procrastination. A large number of studies have confirmed that self-control plays a stable mediating role between time management disposition, perfectionist personality, and physical activity and procrastination behavior. The self-control model believes that the individual’s psychological resources are limited, and self-control depends on limited cognitive resources (Tan et al., 2025), which is related to the individual’s ability to overcome temptations and adhere to goals. Self-control is crucial in this study and is regarded as a key mediator variable that can explain a variety of social behaviors (Ciurbea et al., 1978), its mechanism of action also shows multi-path and multi-level characteristics, for example, in the pursuit of goals, self-control can not only directly promote the achievement of goals, but also be achieved through indirect paths such as increasing learning behavior (Terni, 2014). In addition, there is a close and two-way positive correlation between positive psychological capital and self-control. Studies have shown that positive psychological capital as a whole and its sub-dimensions can significantly positively predict the level of individual self-control. Individuals with high self-control levels also tend to show higher positive emotions, more optimistic attitudes and stronger self-efficacy, thus significantly promoting the development of their positive psychological capital (Lin et al., 2025). Positive psychological capital provides key internal resources for individuals to implement self-control, and the successful use of self-control can further accumulate and expand these psychological resources. In theory, hope and resilience in positive psychological capital can provide abundant psychological resources for individuals to implement self-control, and the effective use of self-control may be the key to transforming positive psychological capital into practical action and inhibiting procrastination behavior (To et al., 2021).

Positive psychological capital can alleviate negative emotions and provide psychological resource support (To et al., 2021); self-control has been proven to be an important mediating variable between negative emotions, such as stress and anxiety, and procrastination. Clarifying this chain mediating role can deepen the understanding of how to reduce procrastination behavior through physical activity, reveal the relationship from physical activity to positive psychological capital accumulation, and then to the improvement of self-control, and finally achieve the process of reducing procrastination behavior, and provide more targeted and operational empirical basis for colleges and universities. Based on this, this study aims to construct and test a chain mediating effect model to explore whether physical activity can enhance the ability of self-control and ultimately reduce procrastination behavior by enhancing the positive psychological capital of medical postgraduates.

A number of studies have pointed out that physical activity significantly positively predict the level of positive psychological capital of individuals by promoting the accumulation of emotional capital (such as improving self-esteem and self-efficacy) and personal capital (such as cultivating self-discipline and persistence) (Li et al., 2024; Liang and Qin, 2022). According to the energy model theory of self-control, all self-control behaviors consume limited internal resources (Liu et al., 2022), positive psychological capital as a positive psychological resource, contains components such as hope and resilience that can provide energy support for self-control (Jia et al., 2021). In addition, the direct inhibitory effect of self-control on procrastination has a solid theoretical basis and empirical support. A large number of studies have confirmed that there is a stable negative correlation between self-control and procrastination behavior (Aini and Mahardayani, 2011; Asani, 2023). Individuals with strong self-control ability are more able to overcome impulses and manage time, thereby reducing procrastination behavior. In summary, the chain path follows the logic of “physical activity → positive psychological capital → self-control → procrastination behavior”, which is in line with the theoretical framework of the human capital model (Bailey et al., 2015; Bailey et al., 2012), and the correlation between each link is supported by direct empirical evidence.

## 2 Research hypothesis

### 2.1 The relationship between physical activity and procrastination behavior of medical postgraduates

Physical activity can not only improve individual physical and mental health, but also change individual behavior habits and behavior patterns. Procrastination behavior is the behavior of individuals who deliberately do not complete the corresponding tasks on time (Armitage and Conner, 2001). Previous studies have shown that there is a significant negative correlation between physical activity and college students “procrastination behavior, indicating that the more frequent, more regular, and more appropriate the intensity of physical activity, the lower the possibility of college students” procrastination behavior (Jianqin et al., 2018). This negative correlation has been verified in different samples such as junior high school students, as well as in different procrastination fields such as academic procrastination and general procrastination tendency (Ren et al., 2021), the college students are less likely to procrastination behavior while ensuring the intensity of physical activity while ensuring the time and frequency of weekly physical activity (Li et al., 2022).

First of all, there may be a negative correlation between physical activity and procrastination behavior of medical postgraduates. The theory of planned behavior takes the relationship between intention and behavior as the starting point. The core idea is that intention directly determines behavior, and behavioral attitude, subjective norm, and perceptual behavior are predictive factors that determine behavioral intention (Bîlbîie et al., 2021). Therefore, the more positive the individual’s attitude towards the goal, the stronger the subjective norm, the stronger the sense of behavioral control, the stronger the behavioral intention to implement the goal orientation, and the more likely the individual is to practice the behavior (Harris and Rogers, 2023).

Secondly, the theory of planned behavior is also one of the most widely used theories in the study of procrastination behavior. Some scholars (Lin and Xinwen, 2014) used the theory of planned behavior to intervene in the procrastination behavior of college students. Chinese scholars explained the procrastination behavior of college students based on the theory of planned behavior, and put forward the view that there is a correlation between TPB and procrastination (Lin) (Yu et al., 2025). Some studies have found that physical activity can significantly negatively predict the procrastination behavior of college students (Zhuan et al., 2024; Zhao and Kou, 2024). The higher the level of individual physical activity, the lower the probability of procrastination behavior (Li et al., 2022).

Therefore, the study proposes the hypothesis H1: physical activity can directly and negatively predict the procrastination behavior of medical postgraduates.

### 2.2 The mediating effect of positive psychological capital between physical activity and procrastination behavior of medical postgraduates

Positive psychological capital is a high-level concept, which is an individual’s positive psychological development state. It is divided into four parts: self-efficacy, hope, optimism, and resilience (Seo and Ko, 2024).

First of all, positive psychological capital is closely related to physical activity. Research has confirmed that physical activity can significantly improve the level of positive psychological capital of college students (Lin et al., 2025; Wei et al., 2024). Physical activity is an effective way to cultivate the positive psychological capital of medical postgraduates. Regular physical activity is often accompanied by challenging goals. In the process of achieving these goals, individuals will continue to accumulate successful experience to enhance self-efficacy, improve hope level, develop an optimistic attitude, and improve psychological resilience, thereby enhancing their confidence in completing their tasks (Luthans et al., 2006). Further research shows that physical activity is positively correlated with psychological capital and contributes to the construction of positive psychological capital (Li et al., 2024). Therefore, physical activity can be regarded as an important antecedent variable of positive psychological capital.

Secondly, positive psychological capital may negatively affect procrastination behavior. Positive psychological capital is a positive mentality that individuals show in the face of challenges and difficulties (Xu et al., 2024), individuals with high positive psychological capital are more willing to challenge themselves in an uncertain environment, maintain vitality in the process of achieving goals, and be more confident in the face of problems to be solved (Jinping et al., 2019); it has also been found that positive psychological capital can motivate for individuals to achieve their goals (Jang, 2022).

Positive psychological capital transcends human capital and social capital in the traditional sense, emphasizing the influence of an individual’s internal positive psychological resources on behavior. Procrastination behavior of medical postgraduates in the university stage is common and complicated, including external factors such as task characteristics and time management, as well as internal psychology such as self-regulation failure and lack of motivation. Individuals with a higher level of positive psychological capital often show less procrastination behavior, enhance individual confidence in completing tasks, and have an optimistic attitude, which helps to actively interpret the value of tasks. Hope to make people more adept at setting and pursuing goals; resilience helps individuals maintain behavioral consistency in the face of stress and challenges. Therefore, positive psychological capital may reduce procrastination behavior by improving individual cognition and emotion. Based on the above discussion, it can be considered that physical activity may indirectly and negatively affect the procrastination behavior of medical postgraduates by enhancing the positive psychological capital of individuals. The establishment of this intermediary path is conducive to deepening the understanding of the internal mechanism between physical activity and procrastination behavior from the perspective of positive psychology.

Therefore, the study proposes the hypothesis H2: positive psychological capital has a negative mediating effect between physical activity and procrastination behavior of medical postgraduates.

### 2.3 The mediating effect of self-control between physical activity and procrastination behavior of medical postgraduates

Self-control refers to the ability of individuals to overcome immediate impulses, habits, or automated reactions, and consciously regulate their own behavior to meet social norms and long-term goals, thereby regulating their own behavior (Zeng et al., 2025). Self-control is a positive psychological quality that has plasticity for the individual’s life and has a positive leading role in the individual’s cognition, emotion, and behavior. According to the Strength Model of Self-Control, Temporal Motivation Theory, and Conceptual Model of Procrastination Behavior, the study suggests that self-control may be an intermediary variable between physical activity and procrastination behavior.

First of all, there is a close relationship between self-control and physical activity. According to the self-control strength model, although psychological resources will be reduced by self-control, such resources can be regenerated through exercise. Enriching psychological resources can enhance the sense of control and improve the ability to cope with stress, thereby reducing the risk of negative emotions in adverse environments (Hou et al., 2022). Research shows that physical activity can significantly positively predict the ability of self-control (Qiu et al., 2023).

Secondly, self-control is closely related to procrastination behavior. Individuals with higher self-control ability show stronger adaptability to the environment and have healthier physical and mental health. Lack of self-control is prone to procrastination behavior (Telzer et al., 2011). According to the theory of time motivation, poor self-control ability is one of the main factors of procrastination behavior (He et al., 2025). It is mainly manifested in the fact that individuals pay more attention to short-term goals and ignore the possible benefits of long-term goals when making decisions.

Therefore, procrastination is a complex phenomenon of self-control failure in essence, and individuals with low self-control ability are more likely to produce procrastination behavior. Self-control has a negative predictive effect on procrastination behavior (Kühnel et al., 2018).

Therefore, the study proposes Hypothesis H3: self-control has a negative mediating effect between physical activity and procrastination behavior of medical postgraduates.

### 2.4 The chain mediating effect of positive psychological capital and self-control between physical activity and procrastination behavior of medical postgraduates

Positive psychological capital is a comprehensive positive psychological resource, which provides important internal support for the development and improvement of self-control ability (Jia et al., 2021).

First of all, studies have shown that there is a significant positive correlation between positive psychological capital and self-control (Li et al., 2024). The ability of self-control is very important in the academic situation of medical postgraduates, which is directly related to whether students can effectively manage time, resist temptation, start and adhere to learning tasks. Self-efficacy in positive psychological capital can enhance individual confidence in completing tasks. Hope and optimism help to maintain goal orientation and positive expectations in the face of boring or difficult learning tasks. Resilience enables individuals to quickly recover from the temporary failure of self-control. Self-control is an individual’s ability to overcome impulses, regulate emotions, and behaviors to meet long-term goals (Wang and Sun, 2023).

Secondly, studies have shown that self-control has a significant negative predictive effect on procrastination behavior (Gökalp et al., 2022). When individuals have good self-control ability, they can better perform their plans, inhibit distraction, and can still push themselves forward in the face of task aversion, thus effectively reducing procrastination. Therefore, self-control constitutes a key behavioral transformation mechanism from positive psychological capital to specific behavioral outcomes.

Finally, there may be a chain mediating path of “physical activity → positive psychological capital → self-control → procrastination behavior” in the influence of physical activity on procrastination behavior of medical postgraduates. Physical activity promotes the accumulation of positive psychological capital of medical postgraduates, the improved positive psychological capital strengthens the ability of self-control, and the enhanced ability of self-control enables medical postgraduates to manage self-behavior more effectively and inhibit procrastination behavior. Through literature review, it is found that there is a link between physical activity and positive psychological capital, a link between positive psychological capital and self-control, and a link between self-control and procrastination behavior, but there is a lack of in-depth research on the internal relations of the four variables.

Therefore, the study proposes Hypothesis H4: positive psychological capital and self-control have a chain mediating effect between physical activity and procrastination behavior of medical postgraduates.

**Figure 1:**
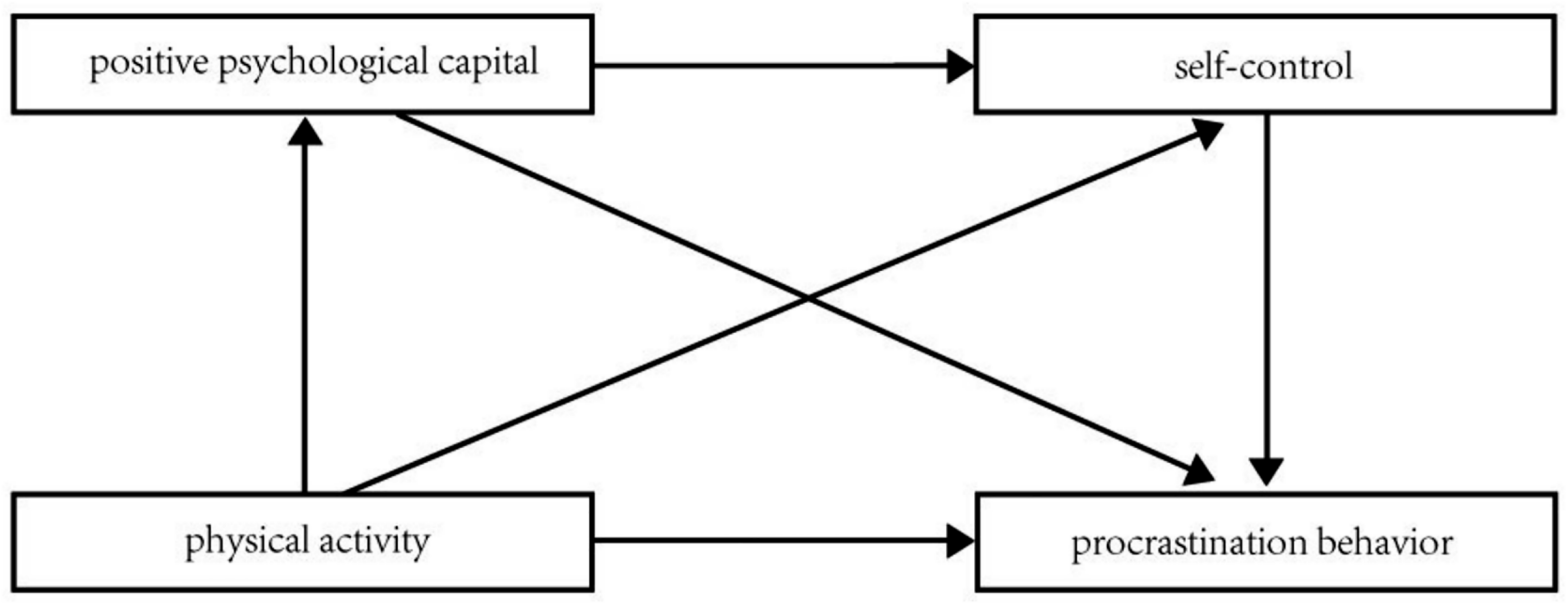
Research hypothesis model.

In summary, the study introduces positive psychological capital and self-control between physical activity and procrastination behavior for the first time, and constructs a chain mediation model, as shown in Figure 1.

## 3 Research objects and methods

### 3.1 Research object

The medical postgraduates from 5 medical universities in Liaoning Province were selected as the survey subjects. The questionnaire follows the principles of voluntary participation, data confidentiality, and anonymous filling out. From October 10, 2025 to December 15, 2025, I made preliminary preparations, including questionnaire sorting, school selection, research process clarification, and research plan design. The questionnaire was distributed and collected on site in December 2025. All subjects are adults. In order to ensure the subjects’ right to know and follow the principle of voluntariness, the informed consent of the subjects was obtained before the test, the content of the survey was kept confidential and the data use was declared. During the test, the counselor organizes to fill in the questionnaire and reminds the students not to miss the information.

A total of 692 questionnaires were collected in this study, and a total of 673 valid questionnaires were collected after eliminating invalid questionnaires, with an effective recovery rate of 97.3%. As shown in Table 1.

**Table 1:** Demographic statistical analysis.

Variable	Gender		Grade		
	Male	Female	Master 1 grade	Master 2 grade	Master 3 grade
Quantity	288	385	249	362	62
Percentage	42.8%	57.2%	37.0%	53.8%	9.2%

### 3.2 Research tools

The “Physical Activity Scale”, “Procrastination Behavior Scale”, “Positive Psychological Capital Scale”, and “Self-Control Scale” were used to measure the psychology of medical postgraduates. In addition, personal basic information includes the gender and grade of medical postgraduates.

#### 3.2.1 Physical activity scale

The level of physical activity of medical postgraduates was measured by the “Physical Activity Scale” compiled by Hashimoto (2005) and revised by Deqing (1994). The “Physical Activity Scale” has three dimensions: exercise intensity, exercise time, and exercise frequency, with one item in each dimension. Using the Likert 5-point scoring method, the two dimensions of exercise intensity and exercise frequency, from “completely inconsistent” to “fully consistent”, were counted from 1 to 5 points, and the exercise frequency dimension from “completely inconsistent” to “fully consistent” was counted from 0 to 4 points.

The overall Cronbach’s *α* coefficient of the “Physical Activity Scale” in the study is 0.832, and the reliability of the scale is good.

#### 3.2.2 Positive psychological capital scale

The “Positive Psychological Capital Scale” compiled by Kuo et al. (2010) was used to measure the level of positive psychological capital of medical postgraduates. There were 26 items, and 26 items were divided into four dimensions: self-efficacy, resilience, hope, and optimism. There are 7 items for self-efficacy and resilience, and 6 items for hope and optimism. Using the Likert 5-point scoring method, from “completely inconsistent” to “completely consistent”, 1 to 5 points are counted, respectively. The average score of each dimension is taken as the score of each dimension, and the average value of each dimension is taken as the total questionnaire score. The higher the score is, the higher the positive psychological capital level of each dimension or the whole is. There are 6 items for reverse scoring.

The overall Cronbach’s *α* coefficient of the “Positive Psychological Capital Scale” in the study is 0.939, and the interval of the four sub-dimensions’ Cronbach’s α coefficient is 0.877–0.936. The reliability of the scale is good.

#### 3.2.3 Self-control scale

“Self-Control Scale” developed by American scholar Tangney et al. (2004) and revised by Shuhua and Yongyu (2008) was used to measure the level of self-control of medical postgraduates. The “self-control scale” consists of five dimensions, namely impulse control, healthy habits, resistance to temptation, focus on work, and control of entertainment, with a total of 19 items. Using the Likert 5-point scoring method, from “completely inconsistent” to “completely consistent”, 1 to 5 points are counted respectively, of which 4 items are positive scoring, and the remaining items are reverse scoring. The higher the total score, the stronger the self-control ability.

The overall Cronbach’s α coefficient of the “Self-Control Scale” in the study is 0.899, and the interval of the Cronbach’s α coefficient of the five sub-dimensions is 0.832–0.869. The reliability of the scale is good.

#### 3.2.4 Procrastination behavior scale

The procrastination behavior of medical postgraduates was measured by the “Procrastination Behavior Scale” compiled by Aitken in 1982 (Xiaoli et al., 2008). “Procrastination Behavior Scale” is a single-dimensional self-rating scale composed of 19 items. Using the Likert 5-point scoring method, 1 to 5 points were scored from “completely inconsistent” to “completely consistent”, of which 9 items were reverse-scoring.

The overall Cronbach’s α coefficient of the “Procrastination Behavior Scale” in the study is 0.884, and the reliability of the scale was good.

### 3.3 Data statistics

Import the valid data sorted into the EXCEL table into SPSS 27.0 software and analyze the data. (1) Descriptive statistics were used for demographic analysis. (2) Cronbach alpha test was used to test the reliability of the scale. (3) Harman single factor test was used to test the common method deviation of the data. Independent sample t-test and single factor variance were used to analyze the physical activity, positive psychological capital, self-control, and procrastination behavior (hereinafter referred to as “four variables”) of medical postgraduates of different genders and different grades. (4) Pearson was used to analyze the correlation of the four variables. (5) Multiple regression analysis of the four variables; using the Process 4.2 macro program, the mediating effect was tested by “Model number 6, Confidence intervals 95, Number of bootstrap samples 5,000”.

The specific settings of the variables analyzed by the Process plug-in are as follows: Set the model as Model 6, X = physical activity, M1 = positive psychological capital, M2 = self-control, Y = procrastination behavior.

## 4 Results and analysis

### 4.1 Common method bias test

First of all, because the data used in this study are derived from the self-reports of the subjects, the common method bias is controlled procedurally by anonymous measurement, random arrangement of the order of the scale, setting up the design of the reverse scoring questions, and other measures in the research survey. Secondly, Harman single factor test was used to test the common method deviation of the data. The results showed that the characteristic roots of 15 factors were greater than 1, and the total variation explained by the unrotated first factor was 23.300%, which was much lower than the critical value of 40% (Podsakoff et al., 2003). Therefore, there is no serious common method bias in the data used in this study, so it meets the statistical requirements.

### 4.2 Difference test of different groups

In order to explore whether there are gender differences in physical activity, positive psychological capital, self-control, and procrastination behavior of medical postgraduates, an independent sample t-test was used for analysis, as shown in Table 2. There was no significant difference between male and female medical postgraduates in physical activity, positive psychological capital, self-control, and procrastination behavior.

**Table 2:** Gender differences between different variables.

Variable	Gender	Gender	M	SD	F	*P*
Physical activity	Male	288	3.42361	1.037597	0.451	0.502
	Female	385	3.41732	1.022966		
Positive psychological capital	Male	288	3.89663	0.656100	1.653	0.199
	Female	385	3.83696	0.686766		
Self-control	Male	288	3.85435	0.555225	0.037	0.849
	Female	385	3.85810	0.548066		
Procrastination behavior	Male	288	3.25822	0.670830	0.026	0.873
	Female	385	3.23582	0.651516		

One-way ANOVA test was used to compare the relationship between physical activity, positive psychological capital, self-control, and procrastination behavior among medical postgraduates of different grades, as shown in Table 3. The medical postgraduates of different grades are not significant in physical activity (*F* = 0.241, *p* = 0.786), positive psychological capital (*F* = 2.218, *p* = 0.110), self-control (*F* = 0.323, *p* = 0.724) and procrastination behavior (*F* = 1.113, *p* = 0.329), indicating that there are no differences in physical activity, positive psychological capital, self-control and procrastination behavior among medical postgraduates of different grades, it shows that there is no significant difference in physical activity, positive psychological capital, self-control and procrastination among medical postgraduates of different grades. Therefore, there is no need to do multiple comparisons. Because ‘One-way ANOVA test’ was not significant (*p* > 0.05), ‘there was no statistical difference between the groups’, no subsequent analysis was required.

**Table 3:** Grade differences between different variables.

Variable	Grade	Quantity	M	SD	F	*P*
Physical activity	Master 1 grade	249	3.44444	1.045843	0.241	0.786
	Master 2 grade	362	3.41621	1.001019		
	Master 3 grade	62	3.34409	1.125896		
Positive psychological capital	Master 1 grade	249	3.79163	0.695636	2.218	0.110
	Master 2 grade	362	3.90714	0.669107		
	Master 3 grade	62	3.88648	0.595128		
Self-control	Master 1 grade	249	3.84020	0.569892	0.323	0.724
	Master 2 grade	362	3.85999	0.553762		
	Master 3 grade	62	3.90153	0.450490		
Procrastination behavior	Master 1 grade	249	3.29486	0.673231	1.113	0.329
	Master 2 grade	362	3.21576	0.628573		
	Master 3 grade	62	3.21986	0.770672		

### 4.3 Descriptive statistics and correlation analysis between variables

In each research variable, the skewness score is −1.508 ~ −0.436, and the kurtosis score is −0.162 ~ 4.174. The study believes that when the absolute value of skewness is less than 3 and the absolute value of kurtosis is less than 10, the sample can be considered to obey the normal distribution. The skewness and kurtosis of the variable data meet the requirements. Therefore, the data basically conforms to the normal distribution and can be used for subsequent analysis. In the correlation analysis, the average score of each variable was used for correlation analysis. The results showed that: (1) Physical activity was significantly negatively correlated with procrastination behavior (*r* = −0.309, *p* < 0.01). Physical activity was positively correlated with positive psychological capital (*r* = 0.414, *p* < 0.01) and self-control (*r* = 0.447, *p* < 0.01). (2) Positive psychological capital was positively correlated with self-control (*r* = 0.526, *p* < 0.01). Positive psychological capital was negatively correlated with procrastination behavior (*r* = −0.328, *p* < 0.01). (3) Self-control was significantly negatively correlated with procrastination behavior (*r* = −0.329, *p* < 0.01). As shown in Table 4.

**Table 4:** Correlation analysis of the four variables.

Variable	M	SD	Physical activity	Positive psychological capital	Self-control	Procrastination behavior
Physical activity	3.42001	1.028488	1			
Positive psychological capital	3.86250	0.673967	0.414^**^	1		
Self-control	3.85649	0.550733	0.447^**^	0.526^**^	1	
Procrastination behavior	3.24541	0.659448	−0.309^**^	−0.328^**^	−0.329^**^	1

^**^At the 0.01 level (two-tailed), the correlation is significant.

### 4.4 The chain mediating effect analysis of positive psychological capital and self-control

There is a significant correlation between the four variables of physical activity, positive psychological capital, self-control, and procrastination behavior, which is in line with the statistical requirements for further analysis of the mediating effect of positive psychological capital and self-control. According to the study of Baron and Kenny (1986), there are three conditions for the establishment of the mediating effect: first, the independent variable is significantly related to the mediating variable; second, the independent variable is significantly related to the dependent variable; third, the mediating variable and the dependent variable are significantly correlated when both the independent variable and the mediating variable enter the regression equation.

Firstly, multiple regression analysis showed that physical activity had a significant negative predictive effect on procrastination (*β* = −0.311, *p* < 0.001). After incorporating variables such as physical activity, positive psychological capital, and self-control into the regression equation, the predictive effect of physical activity on procrastination behavior was still significant (*β* = −0.166, *p* < 0.001); physical activity had a significant positive predictive effect on positive psychological capital (*β* = 0.415, *p* < 0.001); physical activity had a significant positive predictive effect on self-control (*β* = 0.276, *p* < 0.001); positive psychological capital had a significant positive predictive effect on self-control (*β* = 0.412, *p* < 0.001). Positive psychological capital had a significant negative predictive effect on procrastination behavior (*β* = −0.172, *p* < 0.001). Self-control had a significant negative predictive effect on procrastination behavior (*β* = −0.163, *p* < 0.001). As shown in Table 5.

**Table 5:** Regression analysis of the relationship between variables in the model.

Regression equation		Overall fitting index			Significance of regression coefficient		
Outcome variable	Prognosis variate	R	R^2^	F	β	SE	t
Procrastination behavior	Gender	0.315	0.099	24.558^***^	−0.017	0.049	−0.461
	Level				−0.058	0.039	−1.582
	Physical activity				−0.311	0.024	−8.461^***^
Positive psychological capital	Gender	0.423	0.179	48.651^***^	−0.044	0.048	−1.248
	Level				0.079	0.038	2.258
	Physical activity				0.415	0.023	11.855^***^
Self-control	Gender	0.583	0.340	86.196^***^	0.022	0.035	0.704
	Level				0.008	0.028	0.266
	Physical activity				0.276	0.019	7.995^***^
	Positive psychological capital				0.412	0.028	11.879^***^
Procrastination behavior	Gender	0.405	0.164	26.144^***^	−0.024	0.047	−0.671
	Level				−0.038	0.038	−1.062
	Physical activity				−0.166	0.026	−4.069^***^
	Positive psychological capital				−0.172	0.042	−4.009^***^
	Self-control				−0.163	0.052	−3.741^***^

^***^p<0.001.

Secondly, the mediating effect test. The PROCESS 4.2 macro program in SPSS 27.0 software was used to control demographic variables (gender and grade), select 5,000 Bootstrap samplings, and test the mediating effect of positive psychological capital alone, the mediating effect of self-control alone, and the chain mediating effect of positive psychological capital and self-control at the 95% confidence interval, as shown in Table 6. The mediating effect includes three indirect effect paths: (1) indirect effect 1 (−0.046) through the way of “physical activity→ positive psychological capital→ procrastination behavior”, accounting for 23.23% of the total effect; the 95% confidence interval does not contain 0, indicating that the indirect effect of the mediating variable is significant. (2) Indirect effect 2 (−0.029) through the way of “physical activity→ self-control→ procrastination behavior”, accounting for 14.65% of the total effect; 95% confidence interval does not contain 0, indicating that the indirect effect of mediating variables is significant. (3) Indirect effect 3 (−0.018) through the way of “physical activity→ positive psychological capital→ self-control→ procrastination behavior”, accounting for 9.09% of the total effect; the 95% confidence interval does not contain 0, indicating that the indirect effect of the mediating variable is significant.

**Table 6:** Bootstrap mediating effect analysis.

Effect situation	Effect	BootSE	BootLLCI	BootULCI	Relative mediating effect
Total effect	−0.198	0.024	−0.244	−0.152	
Direct effect	−0. 105	0.026	−0.156	−0.054	53.03%
Total indirect effect	−0. 093	0.014	−0.122	−0. 066	46.97%
Indirect effect 1 (Ind1): physical activity→ positive psychological capital→procrastination behavior	−0. 046	0.012	−0. 071	−0. 024	23.23%
Indirect effect 2 (Ind2): physical activity→self-control→procrastination behavior	−0. 029	0.008	−0. 046	−0. 014	14.65%
Indirect effect 3 (Ind3): physical activity→ positive psychological capital→ self-control→ procrastination behavior	−0. 018	0.006	−0. 030	−0. 008	9.09%

## 5 Discussion

This study reveals the chain mediating role of positive psychological capital and self-control in the process of physical activity affecting the procrastination behavior of medical postgraduates. The results show that the total effect value of physical activity on procrastination behavior is −0.198, the direct effect value is −0.105, and the total indirect effect value is −0.093. Both the individual mediating effect and the chain mediating effect of positive psychological capital and self-control reach the statistical significance level. The results show that the chain mediation model of the research hypothesis is established, and the four hypotheses are verified.

In addition, this study found no significant differences in the four variables of gender and grade. First of all, the specific sample (geographical, etc.) of this study may have high homogeneity, or its environmental factors (such as uniform curriculum requirements, campus culture) weaken the gender or grade differentiation patterns that are usually observed. Second, the measurement tools or operational definitions of specific variables used in the study may differ from studies that reported differences, thus affecting the detection of differences. This may also suggest that in certain areas of behavioral or psychological traits, the influence of gender and grade is not universal or constant, but is regulated by more complex situational or individual factors. Putting these non-significant findings into discussion in this study not only helps to clarify the scope of application of the conclusions of this study, but also promotes future research to further explore the boundary conditions and mechanisms that lead to these differences.

### 5.1 The relationship between physical activity and procrastination behavior

The results show that physical activity has a negative predictive effect on the procrastination behavior of medical postgraduates through the direct path. The direct effect value is −0.105, and the mediating effect is significant. The research hypothesis 1 is established.

The theory of planned behavior provides a framework for understanding procrastination behavior. The theory holds that behavioral intention is the most direct factor affecting behavior, and behavioral intention is determined by the attitude of medical postgraduates to behavior, subjective norms and perceptual behavior control (Bosnjak et al., 2020). Applying this theory to the physical activity and procrastination behavior of medical postgraduates can be deduced that if medical postgraduates actively participate in physical activity and feel encouragement from tutors, classmates or campus culture, their procrastination behavior will be inhibited. The internal mechanism may be that physical activity can significantly enhance the self-control ability of medical postgraduates, and self-control resources are limited. The self-control ability strengthened by physical activity can be transferred to the self-regulation of study and work. Medical postgraduates who develop the habit of physical activity can not only better plan exercise time, but also generalize this improved self-control ability to professional fields such as learning and scientific research, so as to more effectively manage time, resist distractions, and persist in completing tasks. Reduce the risk of low learning efficiency and insufficient work input caused by delays (Gillebaart and Adriaanse, 2017). On the contrary, if there is a long-term lack of physical activity, medical postgraduates may miss the opportunity to improve their self-control resources and functions, and are more likely to be difficult to concentrate due to ego depletion in the face of heavy academic work and future clinical or field work.

Therefore, it is considered that when medical postgraduates participate in physical activity, the longer the time, the greater the intensity, and the higher the frequency of physical activity, the less likely they are to delay. If medical postgraduates lack physical activity, the performance of procrastination is more obvious, which will lead to medical postgraduates in learning, as well as in the future clinical or field work can not invest enough energy and it is difficult to detect errors in time. In addition, when medical postgraduates develop the habit of physical activity, they can better plan the time of physical activity and enhance their self-control ability, and then transfer this control to all aspects of medical postgraduates. Reduce the risk that they are unwilling to learn professional knowledge and do not work seriously due to procrastination. Therefore, universities and teachers should pay attention to the impact of physical activity on medical postgraduates, and adopt personalized strategies to reduce the procrastination behavior of medical postgraduates through different types of physical activity. First of all, universities and teachers should systematically publicize the benefits of physical activity to improve cognitive function, relieve stress and improve mood through cognitive intervention, and correct the misunderstanding of “exercise delays learning, “so as to shape their positive attitude towards physical activity (Hao et al., 2023). Secondly, universities and teachers should provide structured support, incorporate physical activity into training programs or provide credit incentives, and establish a counseling and support system provided by sports experts, tutors or senior medical postgraduates to help medical postgraduates develop personalized exercise plans. Finally, we should create a positive campus sports culture and environment, organize diversified sports associations and competitions, provide convenient and high-quality sports facilities (Connor, 2016).

### 5.2 The mediating role of positive psychological capital

The results show that positive psychological capital has a negative mediating effect between physical activity and procrastination behavior, the effect value is −0.046, and the mediating effect is significant. The research hypothesis 2 is established.

From the perspective of resource protection theory, the scholars believe that individuals have the basic motivation to acquire and protect important resources (such as positive psychological capital), and resource gain promotes adaptation (Avey et al., 2010; Merino Rivera et al., 2019). This perspective helps to understand how positive psychological capital plays an intermediary role between variables from the perspective of resource dynamics. Positive psychological capital is a high-order compound resource, which maintains the dynamic balance of individuals through pressure adjustment and resource compensation mechanisms. However, in the context of long-term exposure to cumulative ecological risks, individuals may fall into a spiral of resource loss, that is, the continuous erosion of multiple risk factors gradually depletes their positive psychological capital reserves, resulting in a decline in coping efficiency in the face of new stressors (Hobfoll, 2001). This dynamic depletion mechanism causes individuals to fall into a cycle of resource exhaustion and increasing vulnerability, inducing a compensatory collapse of the psychological resource system (Liu et al., 2023). In addition, studies have found that the superposition of cumulative ecological risks will lead to the double depletion of positive psychological capital. First, in the horizontal dimension, the synergy of multi-system risk factors such as family, school and classmates leads to the overload of psychological resources to deal with risk factors; second, in the vertical dimension, the exposure of multi-system risk factors will lead to the progressive failure of positive psychological capital (Hong et al., 2023), that is, when the accumulation of ecological risks breaks through the individual carrying threshold, it will trigger the imbalance between the supply and consumption of positive psychological capital, resulting in defects in the individual psychological regulation system, and ultimately increasing the probability of procrastination behavior of medical postgraduates. Specifically, positive psychological capital is a positive psychological state. Medical postgraduates with higher levels of positive psychological capital have more sufficient psychological resources in the face of stress, and it is easier to help medical postgraduates have a positive psychological state, thus improving their self-control ability. Medical postgraduates with a low level of positive psychological capital are more likely to have negative emotions and reduced self-control ability, thus increasing procrastination behavior.

### 5.3 The mediating role of self-control

The results show that self-control has a negative mediating effect between physical activity and procrastination behavior, the effect value is −0.029, and the mediating effect is significant. The research hypothesis 3 is established.

Regular physical activity can promote individual conscious control of their own behavior, thereby enhancing the ability of self-control. There have been experiments in which subjects were sedentary first and then engaged in aerobic exercise. The experimental results show that the ability of self-control of the subjects was significantly improved, indicating that aerobic exercise can improve the ability of self-control (Muraven, 2010). In addition, procrastination behavior is affected by individual cognition, emotion, personality, behavior and other factors, the Self-regulatory Failure Theory of procrastination behavior believes that self-control is an important buffer factor for procrastination behavior (Rebetez et al., 2016), if medical postgraduates often participate in physical activity and grasp the time, intensity and frequency of exercise, the ability of self-control will be improved. From the academic point of view, medical postgraduates with strong self-control can arrange their learning time more reasonably, so as to avoid procrastination behavior.

### 5.4 The chain mediating effect of positive psychological capital and self-control

The results show that the total predictive effect of physical activity on procrastination behavior is −0.198, and the direct effect is −0.105, the total indirect effect value is-0.093, and the mediating effect is significant. The research hypothesis 4 is established.

In this study, self-control is included as the core protective factor of procrastination behavior, and its theoretical foundation can be traced back to the self-control strength model and resource conservation theory. Medical postgraduates have the basic motivation to acquire, retain and protect their precious resources, which include both external conditions and internal resources such as psychological energy and positive emotions (Hobfoll, 1989; Sonnentag and Fritz, 2007).

The role of physical activity in promoting self-control is achieved through positive psychological capital. From the perspective of self-determination theory, it is explained that satisfying the autonomy, ability and relevance needs of medical postgraduates is the key to stimulating intrinsic motivation and promoting psychological growth (Singh, 2013). Positive psychological capital, as a high-level construct, integrates the four synergistic psychological resources of self-efficacy, hope, resilience and optimism, and constitutes a more powerful and predictable core positive psychological factor (Santana-Cárdenas et al., 2018). Therefore, physical activity systematically cultivate the positive psychological capital of medical postgraduates by meeting the basic psychological needs, which constitutes the primary link of the chain intermediary process. Then, the strengthened positive psychological capital provides continuous support for self-control, so that medical postgraduates can effectively mobilize cognitive resources and maintain goal orientation in the face of negative emotions that cause procrastination behavior, thus showing stronger self-control ability. Finally, self-control ability directly undertakes the function of resisting procrastination behavior through the influence of positive psychological capital. Medical postgraduates with high self-control are better at using cognitive strategies, so that they can better set clear learning plans, manage time, and eliminate interference, thereby significantly reducing non-essential delays in task initiation and execution. The whole chain path of “physical activity → positive psychological capital → self-control → procrastination behavior” has not only been supported by relevant theories, but also its internal mechanism is in line with the rigorous empirical research paradigm.

Positive psychological capital is an integrated positive psychological resource pool, the four core components of self-efficacy, hope, resilience and optimism work together to provide sufficient psychological resources for the will of medical postgraduates in the process of performing tasks (Sood and Puri, 2023), it makes medical postgraduates more likely to maintain goal orientation by mobilizing their psychology in the face of negative emotions or external temptations caused by tasks, thus showing stronger self-control ability (Jia et al., 2021). Specifically, a high level of positive psychological capital provides a stable belief in self-control. Therefore, in the impact of physical activity on procrastination behavior, positive psychological capital has become the primary intermediary link. The role of positive psychological capital is to transform the physiological and behavioral benefits of physical activity into a positive psychological state that is more conducive to self-regulation.

After self-control is strengthened by positive psychological capital, it then takes on the function of resisting procrastination behavior. Self-control is essentially the ability of individuals to consciously suppress immediate gratification and regulate their behavior to serve long-term goals when long-term goals and immediate impulses conflict (Sümer and Büttner, 2022). In the chain mediating model constructed in this study, self-control is the mediating variable of the terminal. Medical postgraduates with a high level of self-control are better at setting clear learning goals, managing time, and eliminating interference, thus significantly reducing the delay in task initiation and execution. In summary, the chain mediation model constructed in this study deepens the theoretical understanding of physical activity on procrastination behavior, and also provides a precise intervention path for alleviating the procrastination behavior of medical postgraduates by designing comprehensive intervention programs.

## 6 Limitations and future directions

The population of this study is medical postgraduates, and the variables studied may also have similar relationships in other student groups. It is suggested that follow-up scholars can consider expanding the scope of the study population in future research. Follow-up studies should continuously improve research methods, include more examination variables and broaden research perspectives, and conduct in-depth research on the root causes and formation mechanisms of procrastination behavior. In addition, the research data are only from five medical universities in Liaoning Province, China. Previous studies have shown that there are significant geographical differences in the level of physical activity among residents in different provinces in China, and the level of physical activity is also statistically significant. Therefore, the findings of this study in Liaoning Province may not be directly extended to other parts of China (such as eastern coastal or western inland provinces with different economic structures and education models). Future research needs to be validated in a wider geographical area and more representative samples.

## 7 Conclusion

The study clarifies the relationship between physical activity, procrastination behavior, positive psychological capital, and self-control. Specifically, physical activity can significantly and negatively predict procrastination behavior; positive psychological capital has a separate mediating effect between physical activity and procrastination behavior of medical postgraduates, and self-control has a separate mediating effect between physical activity and procrastination behavior of medical postgraduates; positive psychological capital and self-control play a chain mediating role between physical activity and procrastination behavior of medical postgraduates. In addition, the study provides a basis for an in-depth understanding of the relationship between physical activity and procrastination behavior of medical postgraduates, and provides a theoretical reference for improving the positive psychological capital and self-control of medical postgraduates and reducing the procrastination behavior of medical postgraduates. Future research should continue to emphasize the key role of physical activity in the overall physical and mental health of medical postgraduates, and conduct longitudinal research to explore the time, frequency, and intensity of the best physical activity.

## Data Availability

The original contributions presented in the study are included in the article/supplementary material, further inquiries can be directed to the corresponding author.
